# CD11c^+^ B Cells Are Mainly Memory Cells, Precursors of Antibody Secreting Cells in Healthy Donors

**DOI:** 10.3389/fimmu.2020.00032

**Published:** 2020-02-25

**Authors:** Marie-Laure Golinski, Mélanie Demeules, Céline Derambure, Gaetan Riou, Maud Maho-Vaillant, Olivier Boyer, Pascal Joly, Sébastien Calbo

**Affiliations:** ^1^INSERM U1234, Normandy University, Rouen, France; ^2^Department of Dermatology, Rouen University Hospital, Rouen, France; ^3^INSERM U1245, Normandy University, Rouen, France; ^4^Department of Immunology, Rouen University Hospital, Rouen, France

**Keywords:** human, CD11c, B cells, microarray, plasma cells, pemphigus

## Abstract

CD11c^+^ B cells have been reported to be increased in autoimmune diseases, but they are detected in the blood of healthy individuals as well. We aimed to characterize CD11c^+^ B cells from healthy donors by flow cytometry, microarray analysis, and *in vitro* functional assays. Here, we report that CD11c^+^ B cells are a distinct subpopulation of B cells, enriched in the memory subpopulation even if their phenotype is heterogeneous, with overexpression of genes involved in B-cell activation and differentiation as well as in antigen presentation. Upon activation, CD11c^+^ B cells can differentiate into antibody-secreting cells, and CD11c could be upregulated in CD11c^−^ B cells by B-cell receptor activation. Finally, we show that patients with pemphigus, an autoimmune disease mediated by B cells, have a decreased frequency of CD11c^+^ B cell after treatment, relative to baseline. Our findings show that CD11c^+^ B cells are mainly memory B cells prone to differentiate into antibody secreting cells that accumulate with age, independently of gender.

## Introduction

Integrins are a family of heterodimeric cell adhesion receptors with function in cell migration, survival, and proliferation (1). CD11c (ITGAX gene) is the integrin αX chain protein, which combines with the β2 chain (CD18; ITGB2 gene). The CD11c/CD18 complex binds complement iC3b-coated particles to induce phagocytosis (2), cell adhesion molecules [intercellular adhesion molecule (ICAM)-1, ICAM-2, ICAM-4, and vascular cell adhesion molecule 1] (3, 4), lipopolysaccharide (5), and collagen and fibrinogen (6, 7). CD11c is abundantly expressed on monocytes, granulocytes, on tissue macrophages, on a subset of dendritic cells, and at low level on neutrophil, but can be expressed as well on a subset of B cells, T cells, and NK cells, with expression level varying from dim to bright (8).

CD11c^+^ B cells are reported to be increased in autoimmune diseases as rheumatoid arthritis (RA) (9), Sjogren's syndrome (10), multiple sclerosis (11), and systemic lupus erythematosus (SLE) (12), or after malarial infection (13). CD11c^+^ B cells accumulate with age in mice and in women with RA and are thus known as “age-associated B cells” (ABC) (9). However, CD11c^+^ B cells are detected in young healthy donors (HD) as well (14). This broad range of conditions could reflect their state of activation depending on the disease or age and might explain why cell surface expression of CD11c^+^ B cells has been reported to be heterogeneous, corresponding to: CD27^+^ memory cell (9), double-negative (DN) IgD^−^CD27^−^ (13), CD21^low^ (10), and T-bet^+^ (12, 15) or T-bet^−^, at least in mice (16). In addition, we reported previously that following toll-like receptor 9 (TLR9) and B-cell receptor (BCR) stimulation, CD11c^+^ B cells from HD were unable to secrete interleukin (IL)-10 (14), while IL-10 is one of the highest messenger RNA (mRNA) expressed found in CD11c^+^ B cells from SLE patient (12).

In this study, we compared the phenotype, frequency depending on HD's age, gene expression profile, mRNA cytokine expression, and capacity of CD11c^+^ vs. CD11c^−^ B cells to differentiate into plasma cells. Despite the fact that CD11c expression was detected in all B cell subpopulations, our findings demonstrate that CD11c^+^ B cells are enriched in memory B cells, which explains their strong ability to differentiate into antibody-secreting cells. Moreover, we show that CD11c upregulation is controlled by BCR stimulation. We report an increase in the frequency of CD11c^+^ B cells from blood in Pemphigus, an autoantibody-mediated disease targeting the skin and the mucous membranes, which return to HD's level following treatment.

## Materials and Methods

### Phenotyping of PBMCs

**Figure 1:**
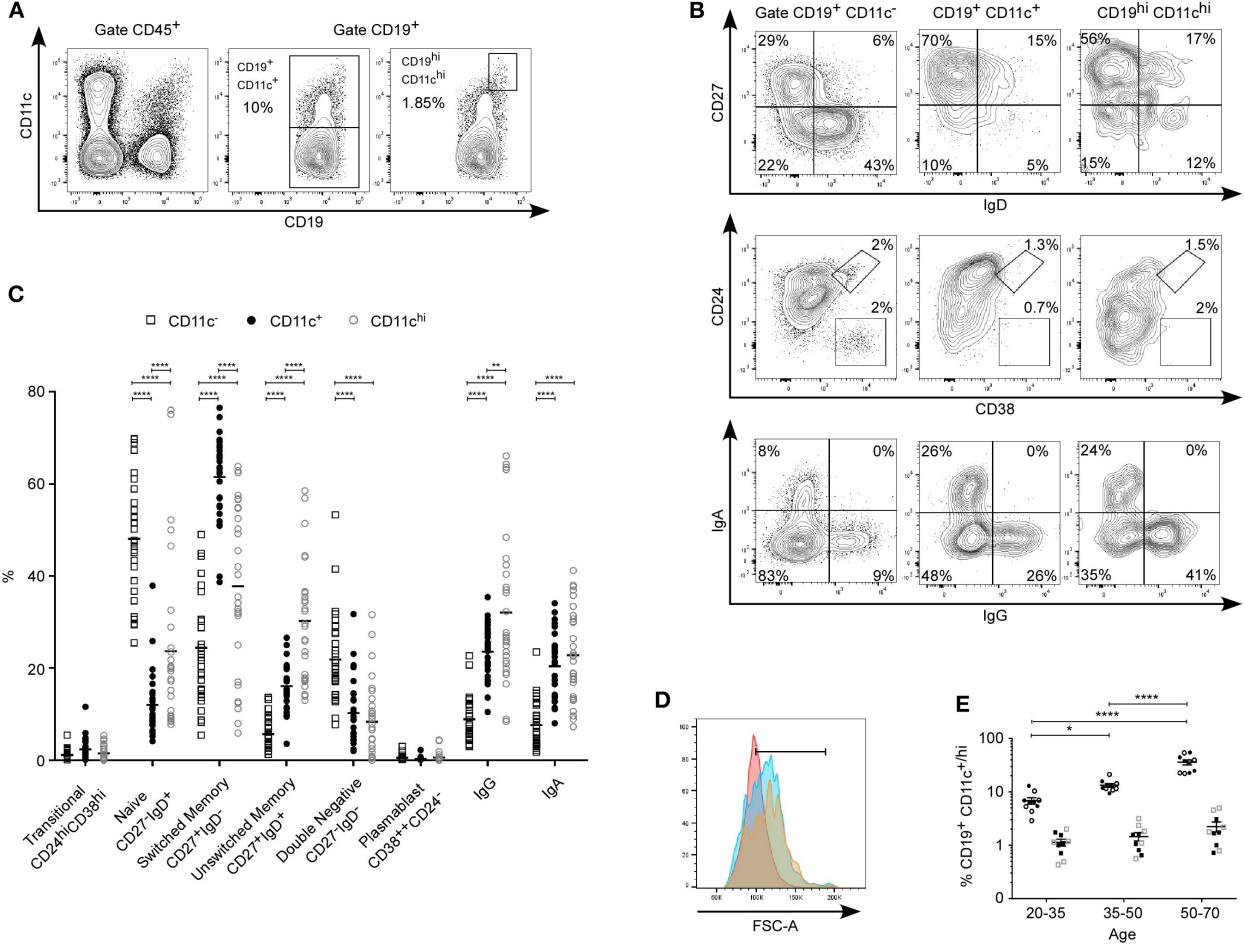
Phenotyping of human CD11c^+^ B cells. **(A)** Expression level of CD11c and CD19 and gating strategy to study CD19^+^CD11c^−^, CD19^+^CD11c^+^, or CD19^+^CD11c^hi^ with one representative frequency. **(B)** CD27, IgD, CD24, CD38, IgA, and IgG expression on CD19^+^CD11c^−^, CD19^+^CD11c^+^, or CD19^+^CD11c^hi^ with one representative frequency, and **(C)** proportion of transitional B cells, naive, switched memory, unswitched memory, double negative, plasmablast, IgG^+^, and IgA^+^ for *n* = 30 healthy donors. **(D)** Forward scatter histogram overlay for CD19^+^ CD11c^−^ (pink), CD11c^+^ (blue), and CD11c^hi^ (yellow). The gate is used to determine the Geo mean fluorescence intensity, which is 0.98, 10.8, and 11.3 × 10^4^, respectively. **(E)** Percentage of CD11c^+^ and CD11c^hi^ B cells for donors between age 20 and 35, 35 and 50, and 50 and 70 years old (*n* = 10 for each group; circle = CD11c^+^ B cells, square = CD11c^hi^ B cells, open symbol = woman, fill symbol = man). Significant difference is determined by two-way ANOVA with correction by Sidak's multiple comparison test in **(C)** and with correction by Tukey's multiple comparison test in **(E)**. *p < 0.05, **p < 0.01, ****p < 0.0001. Dot plots from **(A,B)**, and histogram from **(D)** were obtained from a donor age 32.

**Figure 2:**
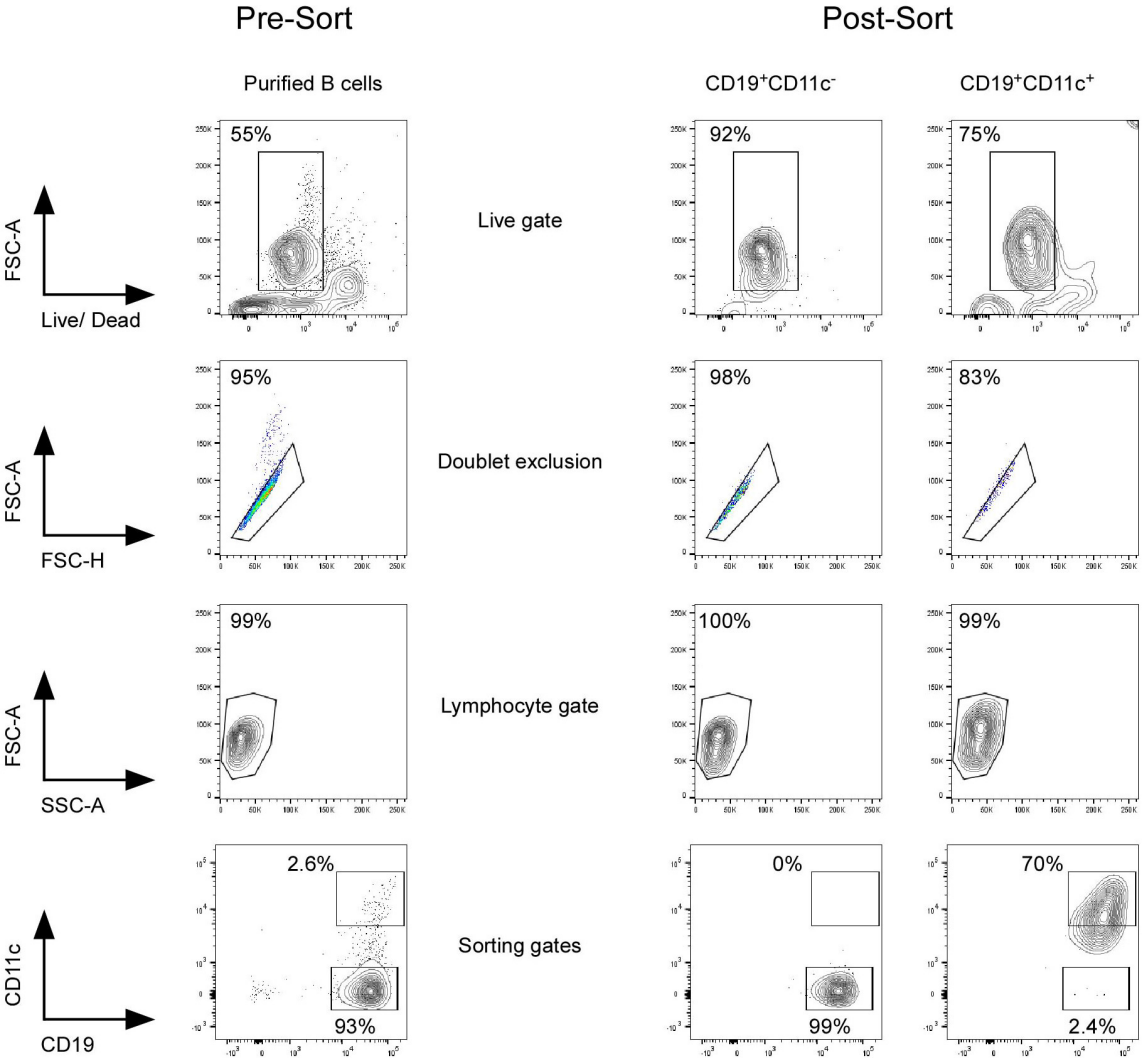
Sorting strategy of CD11c^+^ and CD11c^−^ B cells. Purified B cells by negative selection using magnetic beads were stained with LIVE/DEAD Fixable Aqua Dead Cell Stain Kit, Fc Receptor Blocking Solution, and anti-CD19 and anti-CD11c antibodies. CD11c^+^, including CD11c^hi^ and CD11c^−^ CD19^+^ B cells, were sorted using FACSAria III. Purity of CD11c^+^ and CD11c^−^ B cells was checked after each sorting and was mean 95.3% (±3.4%) and mean 99.3% (±0.4%), respectively. Twenty-six different HD were sorted with age between 20 and 35 years old.

**Figure 3:**
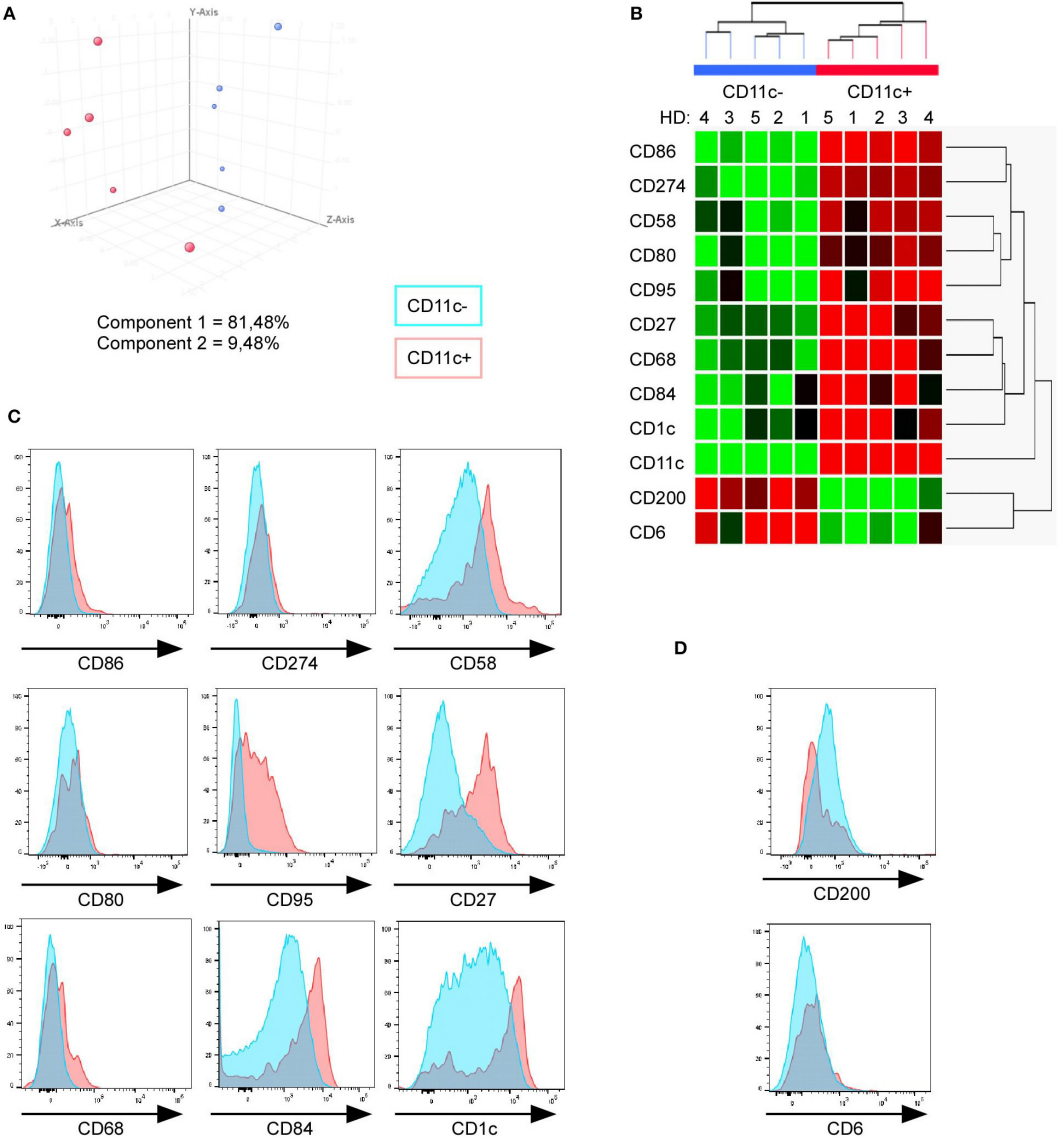
Minimal combination of 12 cell surface markers perfectly discriminate CD11c^+^ from CD11c^−^ B cells. **(A)** Principal component analysis of 10 samples (paired CD11c^+^ and CD11c^−^ B cells from five HD) performed with gene expression level of the 12 mRNA corresponding to cell surface markers among the 512 DEG between CD11c^+^ (red dots) and CD11c^−^ (blue dots) B cells. **(B)** Hierarchical clustering shows that a minimal combination of 12 transcripts is sufficient to perfectly discriminate both populations. **(C,D)** FACS analysis of CD11c^+^, including CD11c^hi^ (red) and CD11c^−^ (blue), was performed to validate this combination of markers at the protein level (one representative result of three independent experiments).

**Figure 4:**
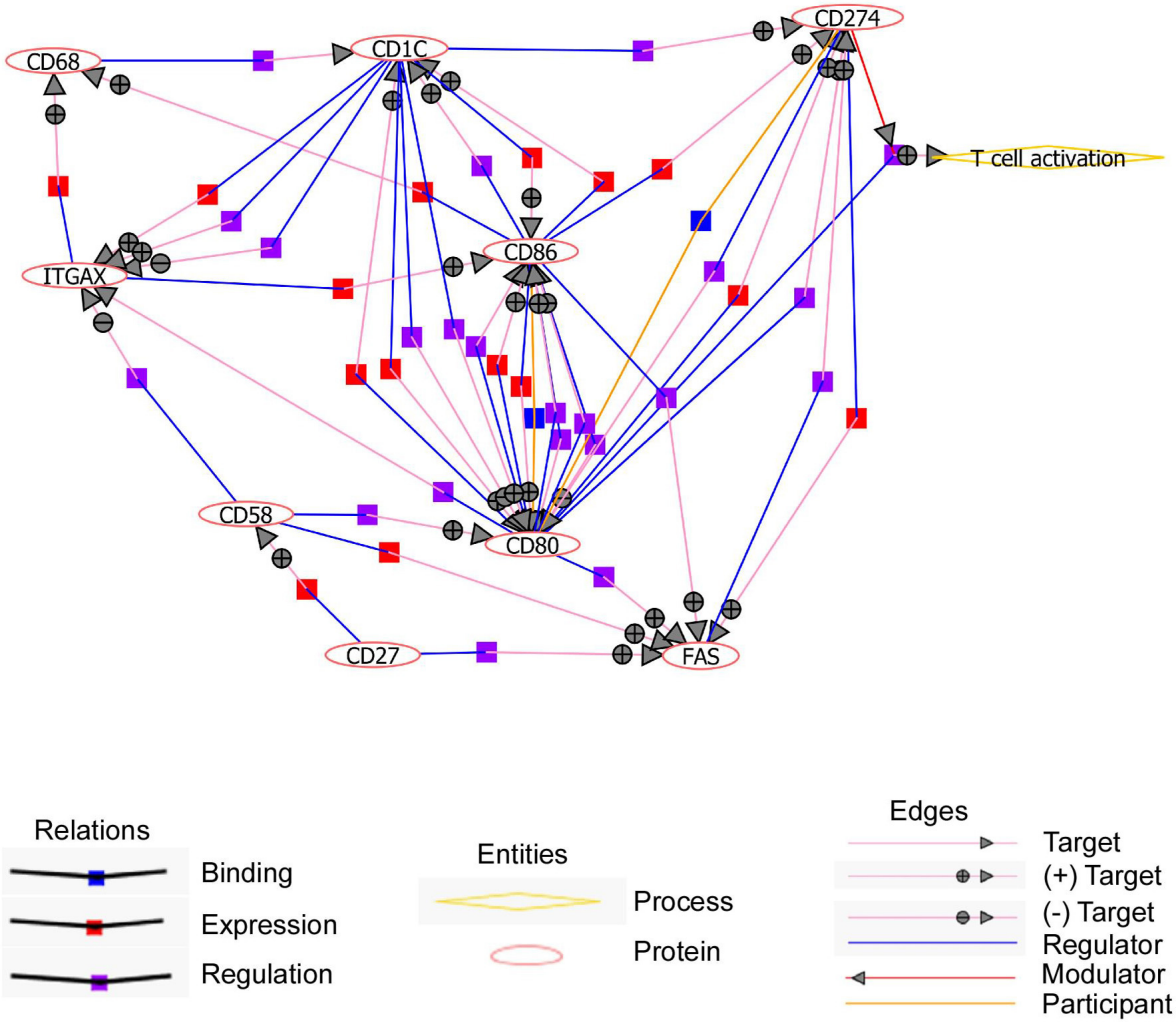
Upregulated cell surface markers on CD11c^+^ B cells interplay to favor T cell activation. Text mining performed with natural language processing (NLP) algorithm shows that 9 out of 12 cell surface receptors are connected all together. Pictograms representing relations, entities, and edges are shown. Circles with + and – symbols represent positive and negative regulations, respectively. Symbols (blue, red, and purple squares) show different modes of relations like binding, expression, and regulation.

**Figure 5:**
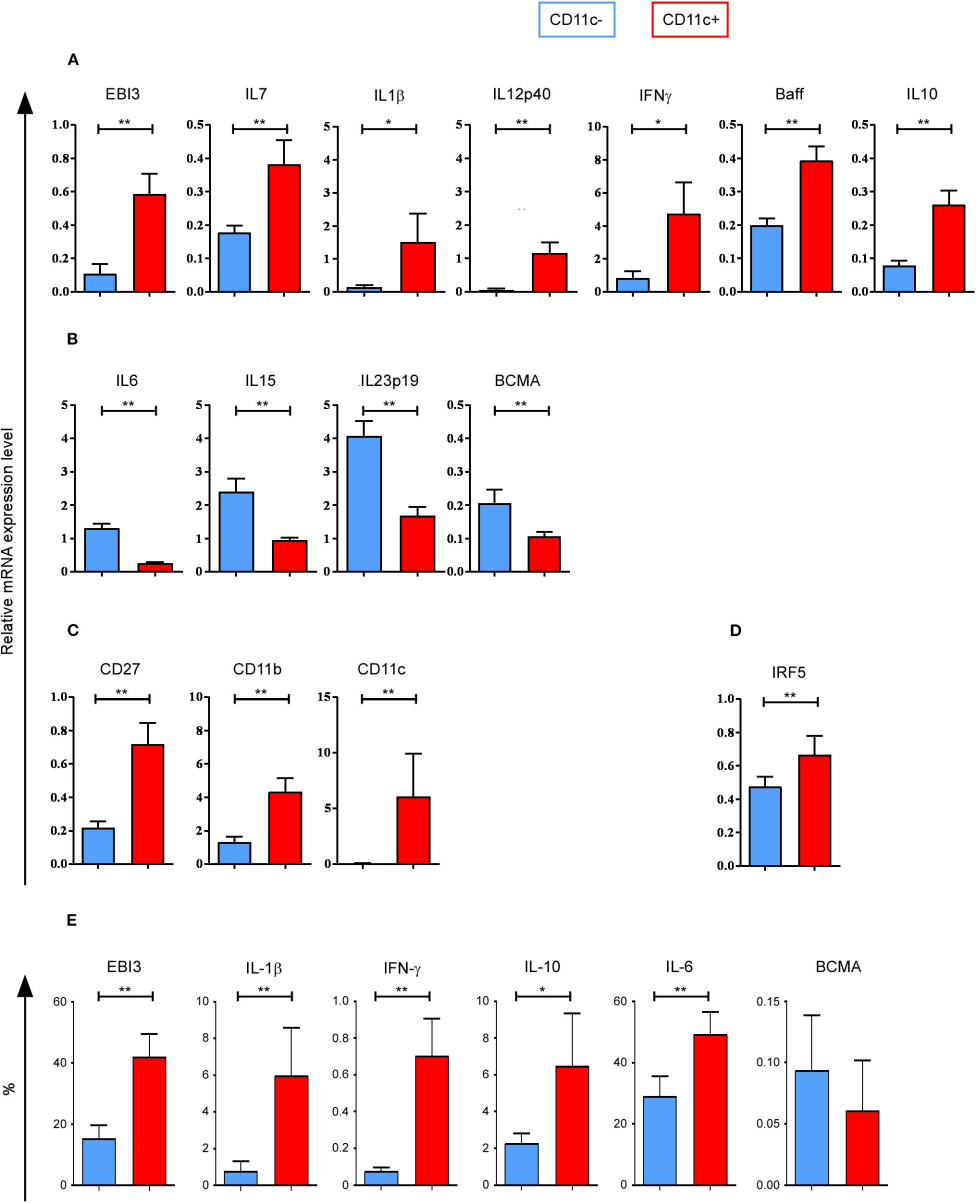
Cytokine profile of CD11c^+^ B cells as well as CD27, CD11b, CD11c, and IRF5 messenger RNA (mRNA) expression. Quantitative PCR (qPCR) analysis of cytokine genes for CD11c^+^ and CD11c^−^ B cells from 10 healthy donors (HD) shows **(A)** upregulated genes and **(B)** downregulated gene expression levels for cytokine genes. **(C)** CD11c^+^ B cells upregulated *CD27, CD11b*, and *CD11c* gene coding for cell surface markers compared to CD11c^−^ B cells. **(D)** *IRF5* gene expression level is shown. **(E)** Peripheral blood mononuclear cells (PBMC) from 10 HD were cultured in the presence of Brefeldin A for 4 h, without any stimulation, and then stained intracellularly with antibodies specific for: Epstein–Barr virus-induced 3 (EBI3), interleukin (IL)-1β, interferon gamma (IFN-γ), IL-10, IL-6, and B-cell maturation antigen (BCMA). Percentage of cytokine expressing cells for CD11c^+^ and CD11c^−^ B cells are shown. Bar graphs show mean ± SEM of relative expression, compared using the Wilcoxon paired *t* test: *p < 0.05; **p < 0.01.

**Figure 6:**
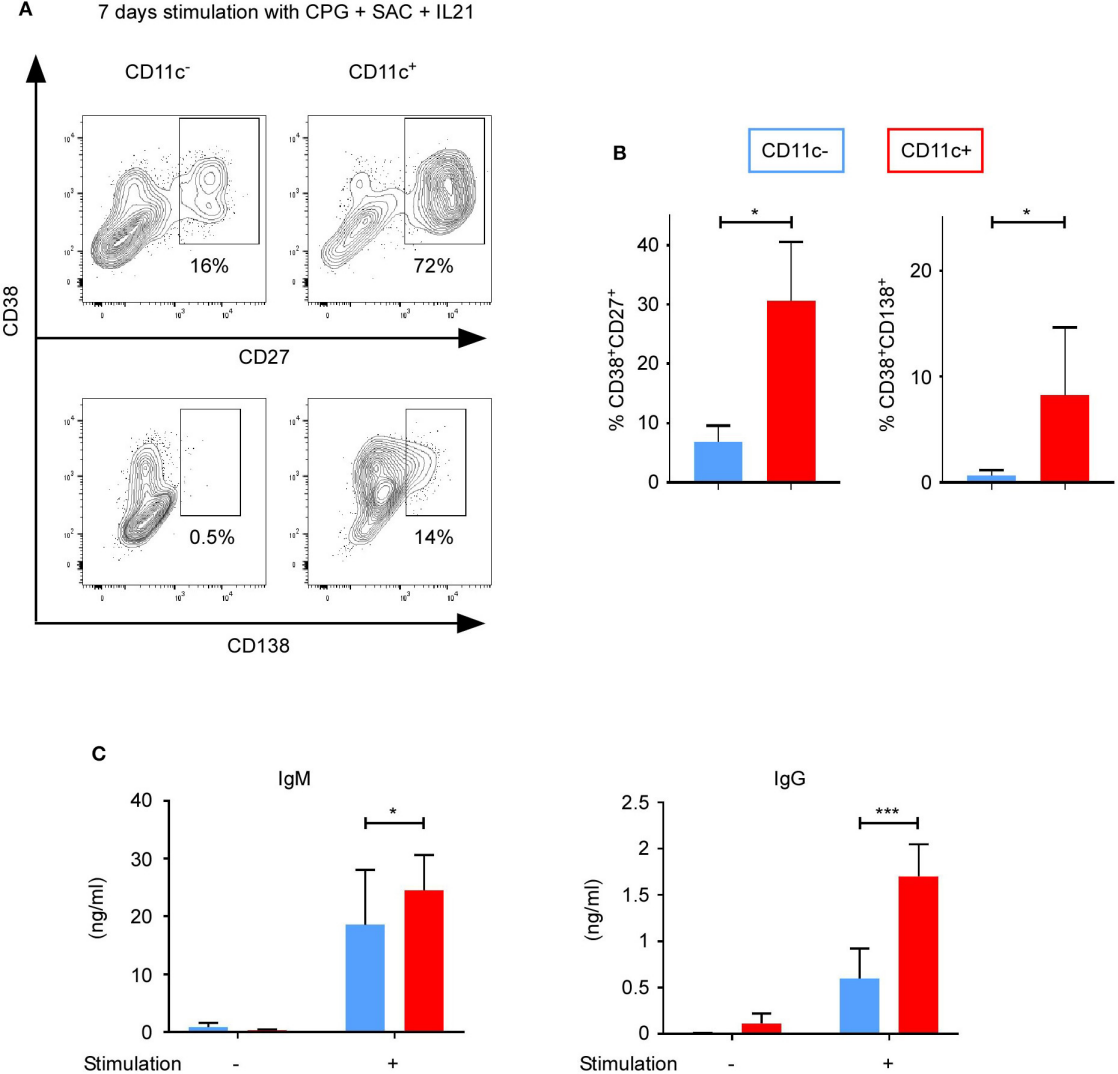
CD11c^+^ B cells differentiate into antibody-secreting cells. Purified CD11c^+^ and CD11c^−^ B cells cultured with CPG + SAC + IL-21. Cells and supernatants were analyzed at day 7. **(A)** Gating strategy to study plasma cells (CD38^+^CD27^+^) and plasmocytes (CD38^+^CD138^+^) (one representative result of three independent experiments is presented). **(B)** Bar graphs show mean ± SEM of % CD38^+^CD27^+^ and % CD38^+^CD138^+^. **(C)** IgG and IgM secretion were measured by ELISA (*n* = 7). Out of seven sorting performed, only three gave us a sufficient number of CD11c^+^ B cells to perform flow cytometry and ELISA analysis. Bar graphs show mean ± SEM of quantity of antibodies (ng/ml). Significant differences are determined using Student's *t* test for **(B)** (*n* = 3; *p < 0.05) and two-way ANOVA with correction by Sidak's multiple comparison test for **C**) *p < 0.05; ***p < 0.001.

**Figure 7:**
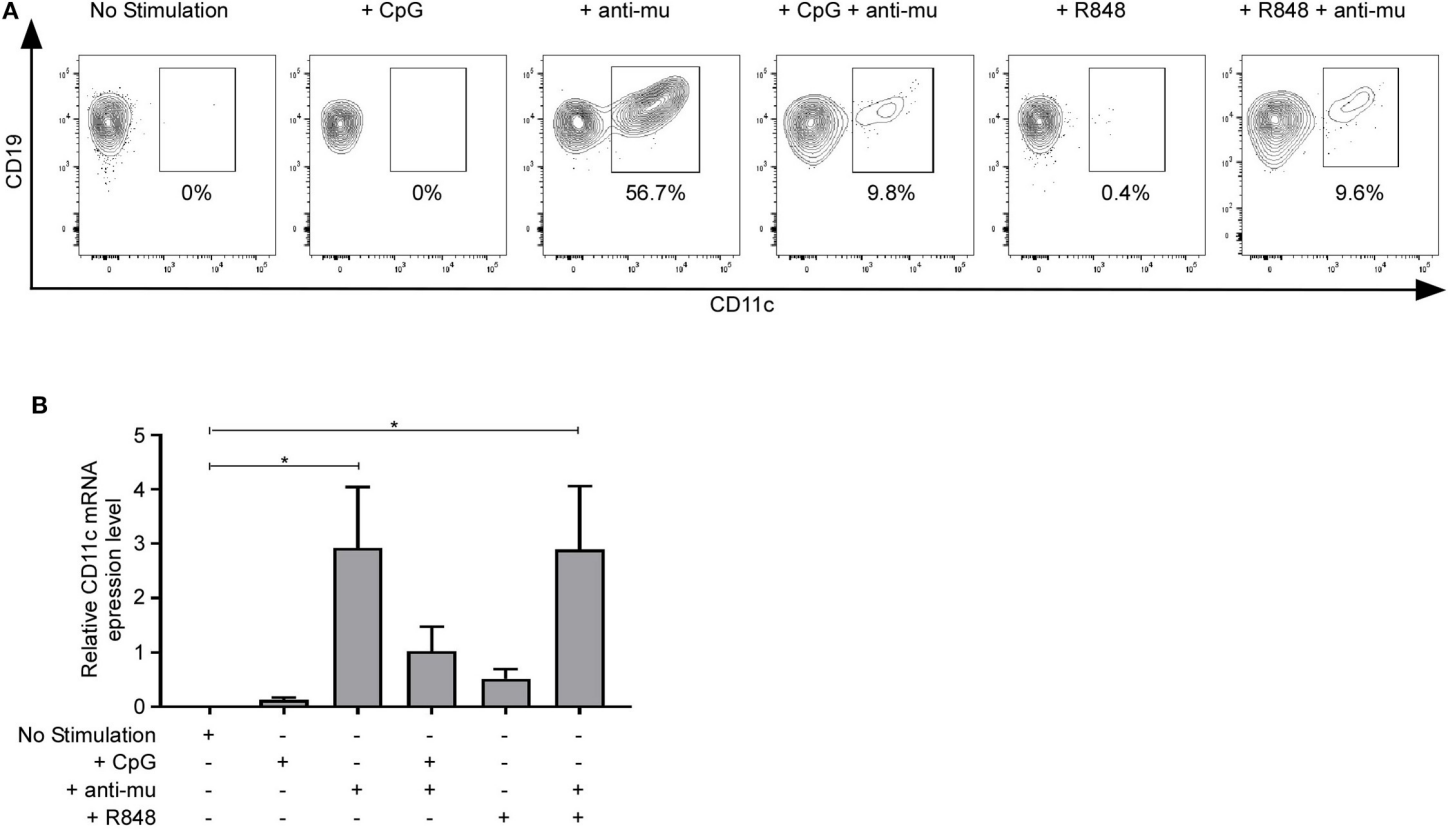
Upregulation of CD11c upon B-cell receptor (BCR) stimulation. CD11c expression was measured after defined stimulation on purified CD11c^−^ B cells by **(A)** flow cytometry (one representative result of three independent experiments is presented) or **(B)** by qPCR (*n* = 4). Bar graphs show mean ± SEM of relative *CD11c* expression. Means were compared using one-way analysis of variance followed by Dunnett *post hoc* test: *p < 0.05.

Peripheral blood mononuclear cells (PBMC) were isolated from buffy coats obtained *via* the French blood bank using Ficoll–Paque density gradient centrifugation (GE Healthcare) (authorization number: PLER-UPR/2018/014). In addition, PBMCs from pemphigus patients from the clinical trial number NCT00784589 were used. This study was approved by the Ethics Committee of the North West in France and conducted according to the Declaration of Helsinki principles. HD age was 20–35 years old, which is the age group that most often donates large volume of blood in our area, unless specified. Representative frequency's examples depicted in Figures 1–7 were obtained from donors in this age group, which is the group of age with the lowest frequency of CD11c^+^ B cells according to Figure 1E.

Phenotype analysis was performed with the cytometer Fortessa^TM^ (Becton Dickinson) using the following markers: LIVE/DEAD® Fixable Blue Dead Cell Stain (Invitrogen), Fc Receptor Blocking Solution (Human TruStain FcX, Biolegend), CD19-PE-Cy7 (clone Hib19, eBioscience), CD11c-PE or APC (clone Bu15, Biolegend), IgA-VioBright-FITC (clone IS11-8E10, Miltenyi), CD27-BV421 (clone M-T271, Becton Dickinson), IgD-AF700 (clone IA6-2, Becton Dickinson), CD38-PerCP-Cy5.5 (clone HIT2, Becton Dickinson), CD24-PE-CF594 (clone ML5, Becton Dickinson), IgG-BV510 (clone G18-145, Becton Dickinson), IgM-BV605 (clone G20-127, Becton Dickinson), CD138-BV711 (clone MI15, Becton Dickinson), CD45-BV785 (clone HI30, Sony), and CD20-APC (clone 2H7, Sony).

To confirm the microarray data, PBMC from five different HD were labeled with the following antibodies: LIVE/DEAD® Fixable Blue Dead Cell Stain (Invitrogen), Fc Receptor Blocking Solution (Human TruStain FcX, Biolegend), CD19-PeCy7, CD11c-PE or APC, CD1c-BV421 (clone L161, Biolegend), CD58-PeCy5 (clone TS2/9, Biolegend), CD84-PE (clone CD84.1.21, Biolegend), CD27-BV421, CD86-BV510 (clone IT2.2, Biolegend), CD95-FITC (clone DX2, Biolegend), CD6-FITC (clone BL-CD6, Biolegend), CD200-BV605 (clone OX104, Biolegend), CD80-BV650 (clone 2D10, Biolegend), CD21-PE (clone HB5, eBioscience) CD274-BV711 (clone 29E.2A3, Biolegend), CD68-PerCP-Cy5.5 (clone Y1/821, Biolegend), IL-27/IL-35 EBI3-PE (clone B032F6, Biolegend), IL-1β-PE (clone AS10, Becton Dickinson), IFN-γ PE (clone 45.15, Beckman Coulter), IL-10 PE (BT-10, Miltenyi), IL-6 PE (clone MQ2-13A5, eBioscience), and BCMA-APC (polyclonal, R&D). All cytometry data were analyzed using FlowJo software (TreeStar Inc). Fluorescence-minus-one controls were used to compensate all flow cytometry data (17).

### Isolation of CD11c^+^ B Cells

B cells were isolated from PBMC using Dynabeads Untouched Human B cells kit (Life Technologies) following the manufacturer's instructions. More than 90% of the isolated cells were CD19^+^. B cells were suspended at 10.10^6^ cells/ml in cold buffer and further stained with LIVE/DEAD Fixable Aqua Dead Cell Stain Kit (Life Technologies), Fc Receptor Blocking Solution (Human TruStain FcX, Biolegend), anti-CD19 Pe-Cy7 (clone HIB19, Biolegend), and anti-CD11c APC (clone BU15, Biolegend). CD11c^+^ and CD11c^−^ CD19^+^ B cells were sorted using BD FACSAria III 4*-*Laser (BD Biosciences). The purity of CD11c^+^ and CD11c^−^ B cells was checked after each sorting and was mean 95.3% (±3.4%) and mean 99.3% (±0.4%), respectively (Figure 2). In total, we have performed 26 cell sorting, from 26 different HD. Owing to the low frequency of CD11c^+^ B cells and the cell sorter's setting to purity (which decreased the yield), B cells were purified from buffy coat, for which the donors are predominantly between 20 and 35 years old in our area. On average, out of 722 ± 301 million PBMC (*n* = 26), we end up with 46 ± 30 million purified B cells that were used for sorting. CD11c^+^ B cells (80,000–1.4 million) were obtained (mean, 5.9 × 10^5^ ± 4.3 × 10^5^ cells, *n* = 26) depending on the donor. Owing to the low number of CD11c^hi^ B cells expected recovery, which represent, on average, 1.6 ± 1.1% of CD19^+^ B cells as shown in Figure 1E (*n* = 30), CD11c^hi^ B cells were included in the sorting of CD11c^+^ B cells.

### *In vitro* Culture of B Cells

CD11c^+^ and CD11c^−^ B cells were cultured at a cell density of 10^6^ cells/ml in Roswell Park Memorial Institute 1640 supplemented with 10% fetal bovine serum, 2 mM l-glutamine, 100 U/ml penicillin, and 100 μg/ml streptomycin (all reagents from Thermo Fisher Scientific). B cells were left unstimulated or stimulated with 3 μg/ml CpG-B 2006 (InvivoGen), 10 μg/ml goat anti-human immunoglobulin A (IgA) IgG IgM (H + L) (anti-Ig; Jackson ImmunoResearch Laboratories) or 1 μg/ml R848 for 48 h at 37°C with 5% CO_2_. For plasmocyte differentiation and antibody production assay, CD11c^+^ and CD11c^−^ B cells were cultured at a cell density of 2.10^5^ cells/ml in the above media and were stimulated with 6 μg/ml CpG-B 2006, 50 ng/ml IL-21, and *Staphylococcus aureus* Cowan I cells (SAC, 1×; Pansorbin cells, Calbiochem) for 7 days at 37°C with 5% CO_2_, unless otherwise stated. Quantity of IgM and IgG secreted were measured by ELISA (READY-SET-GO! Kit, eBioscience). In Figure 5E, PBMC from 10 HD were cultured in 96-well plate U bottom in full media in the presence of Brefeldin A for 4 h, at 2 × 10^6^ cells/ml, without any stimulation. Cells were then stained with Live and Dead, anti-CD45, anti-CD19, and anti-CD11c, washed, fixed, and permeabilized, and stained with different cytokine-specific antibodies that are commercially available. Settings were done using PMA + ionomycin-stimulated cells and fmo.

### RNA Preparation

Total RNAs from CD11c^+^ or CD11c^−^ B cells of 15 HD [5 donors for transcriptome analysis and 10 donors for quantitative PCR (qPCR)] were extracted with RNeasy Mini kit according to the manufacturer's recommendations (Qiagen). The quality and quantity of isolated mRNAs were assessed using the 2100 Bioanalyzer (Agilent Technologies) and the Nanodrop device (Thermo Scientific). All RNA samples had an RNA integrity number of ≥8.2 ± 0.4.

### Microarrays

Whole human genomic DNA microarrays were used to perform one-colored gene expression profiling (4 × 44K Whole Human Genome, Agilent Technologies). One hundred nanogram for each CD11c^+^ or CD11c^−^ B cells sample from 5 HD were labeled by Cyanine-3 according to the manufacturer's instructions (Low Input QuickAmp Labeling Kit, Agilent Technologies). Hybridization was performed at 65°C for 17 h using a hybridization kit (Agilent Technologies). After wash steps, microarrays were scanned with a 5-μM pixel size using the DNA Microarray Scanner GB (Agilent Technologies). Image analysis and extraction of raw data were performed with Feature Extraction Software 10.5.1.1 (Agilent Technologies). Data were normalized using 75 percentile shift and baseline transformed by median of all samples. Only spots that passed these quality controls on 100% of arrays in at least one of two conditions (CD11c^+^ or CD11c^−^) were selected for further analysis. Hierarchical clustering was performed with Pearson coefficient metric and Wards linkage to build the transcripts and sample dendrograms. Data were in agreement with the guidelines for Minimum Information About a Microarray Experiment and were deposited in the database of the National Center for Biotechnology Information's Gene Expression Omnibus (https://www.ncbi.nlm.nih.gov/geo/). Data are accessible using the following accession number: GSE112515. Non-uniform and saturated spots or spots with intensities below the background were not taken into account.

### Functional Analysis of Microarrays

Data from transcriptomic analysis were analyzed with GeneSpring GX V.13.0 (Agilent Technologies). Paired Student's *t* test (*p* < 0.05) with Benjamini–Hochberg correction to check the false discovery rate was used to determine the statistical significance in gene expression levels between CD11c^+^ and CD11c^−^ B cells. The Gene Ontology (GO) analysis was used to investigate the biological processes, molecular function, or cellular localization enriched in the transcripts list showing a significant fluctuation in gene expression between CD11c^+^ and CD11c^−^ B cells. *p* value was computed by standard hypergeometric distribution. The GeneSpring Single Experiment Analysis bioinformatics tool was used for computational analysis to identify potential curated canonical pathways with setting parameters (Reactome and GenMAPP for pathway source), which are enriched in the differentially expressed transcripts list, using WikiPathways database (http://www.wikipathways.org). The significance of the association between the genes and the pathways was measured by Fisher's exact test (minimal number of entities in pathways ≥6 and *p* ≤ 0.001). Text mining was performed with GeneSpring GX V.13.0 (direct interactions with relation score ≥9 and relation types chosen: binding, expression, member, metabolism, promoter binding, protein modification, regulation, and transport).

### Quantitative Real-Time Polymerase Chain Reaction

Three nanograms of each RNA sample were retro-transcribed and preamplified in a 96-well plate containing 0.2 μl Platinum Taq polymerase and SuperScript III reverse transcriptase (Invitrogen), a mixture of Taqman primer probes specific for the transcripts of interest (18), and 5 μl CellsDirect quantitative real-time PCR buffer (Invitrogen). After centrifugation, samples were incubated at 50°C for 15 min for reverse transcription, followed by 20 cycles of 95°C, 15 s and 60°C, 4 min for amplification. Subsequent preamplified complementary DNA (cDNA) was stored at −20°C until analysis. After one-fourth dilution in Tris–EDTA buffer, each cDNA sample was then separated into 48 separate reactions for further qPCR using the BioMark 48.48 dynamic array nanofluidic chip (Fluidigm, Inc.) as described in (19). Wells with no RNA were used as negative controls. Data were analyzed using Real-Time PCR Analysis software with normalization of the Ct value for each gene using the mean of five housekeeping genes (*GAPDH*, β*2-M, TUBB, GUSB*, and *HPRT1*) as calibrator, using geNorm (20). We considered that gene was expressed if Ct value was <40, and expression curve was a sigmoid. To compare expression level between samples, a standard curve was first created with a pool of cDNA from all samples. From this pool, serial dilutions were performed, and qPCR values were assigned arbitrary values (6 points ranging from 100 to 0.4). The gene expression level of samples was obtained using this standard curve.

### Statistical Analyses

All experiments were performed using at least three different cell cultures or blood donors in independent experiments. GraphPad Prism 7 was used to create the figures. Student's *t* test and the Wilcoxon paired *t* test were used to assess normally distributed data and non-normally distributed data, respectively. One-way ANOVA with correction by Bonferroni's posttest, two-way ANOVA with correction by Sidak's posttest, and one-way ANOVA with Dunnett's posttest were used. *p* < 0.05 was considered significant.

## Results

### Phenotyping of Human CD11c^+^ B Cell in Blood of HD

We used HD's blood from the French blood bank, in an age group between 20 and 70 years and with an equivalent number of men and women. Using flow cytometry, expression level of CD11c on blood monocytes and B cells from HD was equivalent, fluctuating from dim to bright (Figure 1A) with percentages of CD19^+^CD11c^+^ varying from 3 to 55% (mean, 19 ± 3, *n* = 30). Among CD11c^+^ B cells, a small subpopulation, bright for CD19 and CD11c, was detected (1.6 ± 0.2%; range, 0.4–5%, *n* = 30). First, CD11c^+^ B cells were compared with CD11c^−^ B cells. We found that CD11c^+^ B cells were significantly enriched in the switched memory population (CD27^+^IgD^−^: 62 ± 2% for CD11c^+^ vs. 24 ± 2% for CD11c^−^, *p* < 0.0001), as in the unswitched memory population (CD27^+^IgD^+^: 16 ± 1 vs. 6 ± 1%, *p* < 0.0001) (Figures 1B,C). In addition, CD11c^+^ B cells had a significantly higher proportion of IgG^+^ and IgA^+^ (IgG^+^, 24 ± 1 vs. 9 ± 1%, *p* < 0.0001; IgA^+^, 20 ± 1 vs. 8 ± 1%, *p* < 0.0001). CD11c^+^ B-cell frequency was reduced in the naive (CD27^−^IgD^+^, 12 ± 1 vs. 48 ± 2%, *p* < 0.0001) and in the DN population (CD27^−^IgD^−^, 10 ± 1 vs. 22 ± 2%, *p* < 0.0001). No significant difference in frequency was observed between CD11c^+^ vs. CD11c^−^ in plasmablast (CD38^++^CD24^−^: 0.3 ± 0.1 vs. 0.5 ± 0.1%) nor in transitional B cell population (CD24^hi^CD38^hi^, 2.3 ± 0.4 vs. 1.1 ± 0.2%; *p* = NS). Second, CD11c^+^ B cells were compared to CD11c^hi^ B cells. Interestingly, the CD11c^hi^ population was enriched in the naive (24 ± 3%, *p* < 0.0001) and the unswitched memory population (30 ± 2%, *p* < 0.0001), and decreased in the switched memory population (38 ± 3%, *p* < 0.0001) (Figures 1B,C), suggesting a retrograde control of CD11c expression during B-cell ontogeny and differentiation. No significant difference was observed between CD11c^hi^ and CD11c^+^ frequencies in the transitional, DN, and plasmablast populations (respectively for CD11c^hi^: 1.5 ± 0.3, 8.4 ± 1.5, and 0.6 ± 0.2%). However, CD11c^hi^ B cells had a significantly higher proportion of IgG^+^ (IgG^+^, 32 ± 2.9%, *p* < 0.01). Of note, CD11c^hi^ B cells had a higher proportion of larger cells than CD11c^+^ and the CD11c^−^ B cells (Figure 1D), suggesting a different stage of differentiation or activation (forward scatter-A geometric mean fluorescence intensity = 111.57, 104.31, 95 × 10^3^ for CD11c^hi^, CD11c^+^, and CD11c^−^ B cells, respectively. *p* < 0.0001, *n* = 30).

Since CD11c^+^CD27^+^ B cells have been described to accumulate in aged women with RA (9), we assessed CD11c^+^ B-cell frequency depending on age and gender. Figure 1E shows that CD11c^+^ B-cell frequency increased with age from 7 ± 1% in HD aged 20–35 years, up to 35 ± 5% in HD aged 50–70 years (*p* < 0.0001), with no difference depending on gender. However, no difference was observed for CD11c^hi^ B-cell frequency between age groups.

Overall, CD11c was expressed in all B cell subpopulations, suggesting a role in all B-cell development steps, with an increased frequency in antigen-driven B cells, which accumulate with age of HD, independently of gender.

### Gene Expression Profile

To better define the cellular characteristics of circulating CD11c^+^ B cells, we then performed a microarray analysis of CD11c^+^ vs. CD11c^−^ B cells from five HD's blood. After negative selection using magnetic bead, purified B cells from buffy coat were sorted based on CD11c expression using BD FACSAria III. Purity of CD11c^+^ and CD11c^−^ B cells was checked after each sorting and was 95.3% (±3.4%) and 99.3% (±0.4%), respectively (Figure 2).

To identify the differentially expressed genes (DEG) between paired CD11c^+^ and CD11c^−^ B cells, total RNA were hybridized on whole human genome microarrays by one-color technology and analyzed with GeneSpring GX V.13.0 (Agilent Technologies). Out of 19,905 transcripts that passed the quality filters after exclusion of spikes and flagged probes, a fold change cut-off of 2 between CD11c^+^ and CD11c^−^ samples identified 1,894 transcripts. To highlight the most discriminant transcripts, we applied a fold change cut-off of 3. After paired *t* test with Benjamini–Hochberg correction for multiple testing (*p* < 0.05), removal of duplicate probes, pseudogenes, and uncharacterized loci, 307 DEG were found to be upregulated and 205 downregulated in CD11c^+^ compared with CD11c^−^ B cells (Supplemental Table 1).

GO analysis was performed with the 512 DEG and identified 145 biological processes, which mainly corresponded to regulation of cell activation (GO:0050865; *p* = 7.44 × 10^−12^), regulation of multicellular organismal process (GO:0051239; *p* = 5.41 × 10^−11^), regulation of immune system process (GO:0002682; *p* = 1.83 × 10^−9^), regulation of cell adhesion (GO:0030155; *p* = 1.49 × 10^−8^), and response to stimulus (GO:0050896; *p* = 2.25 × 10^−9^). To find molecular functions enriched in these DEG set, Wiki and Kyoto Encyclopedia of Genes and Genomes (KEGG) pathway analyses were performed using the Single Experiment Analysis tool from GeneSpring software and DAVID Bioinformatics Resources (21). Pathways related to TLR signaling (WP1449-45049, *p* = 4.2 × 10^−4^; WP75-46210, *p* = 0.001; KEGG 04620, *p* = 0.029), inflammatory response (WP453-41201, *p* = 1.02 × 10^−5^), cell adhesion molecules (KEGG 04514, *p* = 0.021), FoxO signaling pathway (KEGG 04068, *p* = 0.034), and cytokine signaling pathway (KEGG 04060, *p* = 0.04) were listed. These findings indicate that CD11c^+^ B cells have a clear distinctive gene expression profile related to active immune response.

### Differential Expression of CD1c, CD58, CD68, CD84, CD86, CD27, CD274, Fas, and CD200 on Human CD11c^+^ B Cells

Out of 512 DEG, we selected 12 transcripts coding for cell surface receptors. *CD6* and *CD200* were underexpressed, and *CD11c (ITGAX), CD1c, CD84, CD68, CD27, Fas* (CD95*), CD80, CD58, CD274*, and *CD86* were overexpressed in CD11c^+^ B cells vs. CD11c^−^ B cells. Principal component analysis (Figure 3A) and hierarchical clustering (Figure 3B) performed with this minimal combination of 12 transcripts were sufficient to accurately discriminate CD11c^+^ from CD11c^−^ B cells.

We then evaluated by fluorescence-activated cell sorting (FACS) if these 11 markers (excepting CD11c) could discriminate CD11c^+^ from CD11c^−^ B cells as suggested by the microarray data. We found that CD11c^+^ B cells overexpressed CD86, CD58, CD95, CD27, CD68, CD84, and CD1c in comparison with CD11c^−^ B cells (Figure 3C), even if the expression level for these markers was heterogeneous, which might suggest a specific level of expression by the different CD11c^+^ B cell subpopulations. On the other hand, CD200 expression was lower in a fraction of CD11c^+^ B cells compared with CD11c^−^ B cells (Figure 3D). No difference was observed for the CD274, CD80, and CD6 markers, which were weakly expressed by B cells (Figures 3C,D). Overall, the differences in the mRNA expression level between CD11c^+^ and CD11c^−^ B cells were confirmed at the protein level for 8 out of 11 markers, although the expression of these markers was not strictly associated with the CD11c^+^ B cell population. However, text mining from bibliographic data performed with natural language processing algorithm showed that 9 out of 12 cell surface receptors (CD11c, CD1c, CD27, CD58, CD68, CD80, CD86, CD95, and CD274) were related and constituted a functional network associated with CD11c (Figure 4).

### Cytokine Gene Expression of CD11c^+^ B Cells

To characterize the cytokines expressed by CD11c^+^ and CD11c^−^ B cells, we analyzed the expression of 25 cytokines known to be secreted by B cells, in addition to 5 housekeeping genes and 1 transcription factor (19), using Biomark HD real-time PCR. Sorted B cells from 10 HD were analyzed. *EBI3, IL7, IL1*β, *IL12p40, IFN*γ, *Baff*, and *IL10* were found to be significantly upregulated, while *IL6, IL15, IL23p19*, and *BCMA* were found significantly downregulated in *ex vivo* purified CD11c^+^ B cells (Figures 5A,B). qPCR analyses confirmed the significantly different expression of *EBI3* and *IL6* observed using microarray, with a fold change > 3 (Supplemental Table 1) and that of *IL7, IL15*, and *IL23p19* with a fold change > 2 (data not shown). No significant difference in expression of the genes, *April, TNF*α, *IL1RA, IL12p35, TNF*β, *Trail, TGF*β*2, CD19, Taci*, and *BAFF-R*, was observed between CD11c^+^ and CD11c^−^ B cells (data not shown). *IL2, IL5, IL9, IL13, IL17A, IL17F, IL27p28*, and *IL21* mRNAs were not detected by qPCR. To confirm cytokine preferential expression at the protein level, PBMC from 10 HD were cultured in the presence of Brefeldin A for 4 h, without any stimulation, and then stained intracellularly with commercially available antibodies specific for: EBI3, IL-1β, IFN-γ, IL-10, IL-6, and BCMA. We confirmed a significant preferential expression for EBI3, IL-1β, IFN-γ, and IL-10 at the protein level for CD11c^+^ B cells (*n* = 10) and a tendency for a lower expression of BCMA, even if the percentage of stained cells was very low (Figure 5E). However, we observed a higher percentage of IL-6^+^ cells among CD11c^+^ B cells than CD11c^−^ B cells, which is the opposite of what we observed at the RNA level. This discrepancy could be due to the instability of *IL6* mRNA (22) or by a regulatory IL-1β feedback loop as IL-6 is induced by IL1-β (23).

We confirmed that *CD27* was significantly upregulated in CD11c^+^ B cells, as well as *CD11b* and *CD11c* (Figure 5C). The transcription factor *IRF5* was also significantly upregulated in CD11c^+^ B cells (Figure 5D), which was confirmed by microarray data with a fold change > 2 (data not show).

In short, cytokine genes overexpressed in CD11c^+^ B cells corresponded to both pro-inflammatory (IFN-γ, EBI3, IL-1β, IL-12p40) and anti-inflammatory (EBI3, IL-10) cytokines, and cytokines involved in B-cell survival (IL-7, Baff, IL-10), activation, and differentiation (IL-1β, IFN-γ) as well as in isotype switch (EBI3, IFN-γ, and IL-10) (24–26).

### Differentiation of CD11c^+^ B Cells Into Plasma Cells

Since CD11c^+^ B cells were enriched in the memory subpopulation and upregulated IRF5, which is involved in the differentiation into plasma cells (27, 28), we assessed the ability of CD11c^+^ B cells to differentiate into antibody-secreting cells. CD11c^+^ and CD11c^−^ were cultured without or with stimulation (CPG + SAC + IL-21) for 7 days. Plasmablast and plasmocyte differentiation was evaluated by FACS using CD27^+^CD38^+^CD138^−^ and CD27^+^CD38^+^CD138^+^ expression, respectively (Figure 6A) (27). A higher proportion of CD11c^+^ than CD11c^−^ B cells was able to differentiate into plasmablasts (31 ± 10 vs. 7 ± 3%; *n* = 6, *p* < 0.05) and plasmocytes (8 ± 3 vs. 1 ± 0.2%; *p* < 0.04) (Figure 6B). Interestingly, the number of CD11c^−^ B cells upregulating the plasmocyte-specific marker CD138 did not increase, even after a longer stimulation time of 9 days (data not shown). On the opposite, for a shorter stimulation time of 5 days, 29% (±17.1%) CD11c^+^ B cells differentiate into plasmablasts vs. 6.3% (±2.4%) for CD11c^−^ B cells, and 3.1% (±2.7%) CD11c^+^ B cells differentiate into plasmocytes vs. 0% (±0%) for CD11c^−^ B cells (*n* = 3; *p* > 0.3, data not shown).

We then measured the IgM and IgG secretion using ELISA. After 7 days of stimulation, CD11c^+^ B cells secreted 1.3-fold more IgM and 2.8-fold more IgG than CD11c^−^ B cells (Figure 6C) (for IgM from CD11c^+^ B cells, 25 ± 6 ng/ml, and for CD11c^−^ B cells, 19 ± 10 ng/ml, *n* = 7, *p* = 0.011; for IgG from CD11c^+^ B cells, 1.7 ± 0.3 ng/ml, and for CD11c^−^ B cells, 0.6 ± 0.3 ng/ml; *n* = 7, *p* = 0.0002). A 2.7-fold higher secretion of IgM and 1.4-fold higher secretion of IgG by CD11c^+^ B cells than CD11c^−^ B cells was also observed after a shorter stimulation duration of 5 days (IgM, 27.1 ± 10.3 vs. 10 ± 5.8 ng/ml; *p* = 0.014; IgG, 1.4 ± 0.3 vs. 1 ± 0.5 ng/ml; *p* = 0.0093). Without stimulation, neither CD11c^+^ nor CD11c^−^ B cells upregulated CD138 (data not shown) nor produced IgM or significant quantity of IgG (Figure 6C). Therefore, upon stimulation, CD11c^+^ B cells were found prone to differentiate into antibody-secreting cells, especially plasmocytes, with a higher frequency of immunoglobulin class switched, which is probably due to the higher frequency of memory B cells in the CD11c^+^ B cells subpopulation.

### Upregulation of CD11c Upon BCR Stimulation

We next addressed whether CD11c^−^ B cells could upregulate CD11c upon activation. Purified CD11c^−^ B cells were cultured without stimulation or with different stimuli: CpG (TLR9 agonist), anti-Ig, R848 (TLR7/8 agonist) for 48 h. CD11c expression was measured by qPCR and flow cytometry. TLR9 or TLR7 stimulation did not upregulate CD11c, while BCR stimulation upregulated CD11c at the protein and mRNA levels (Figure 7), demonstrating that CD11c upregulation is controlled by BCR stimulation. Interestingly, the combine stimulation TLR + anti-Ig inhibit CD11c upregulation induced by BCR stimulation alone, at the protein and mRNA level for TLR9, while only at the protein level for TLR7. Therefore, CD11c upregulation is controlled by BCR stimulation but can be counteracted by TLR7 or TLR9 costimulation.

### Higher Frequency of CD11c^+^ B Cells in Pemphigus Patients Before Treatment

**Figure 8:**
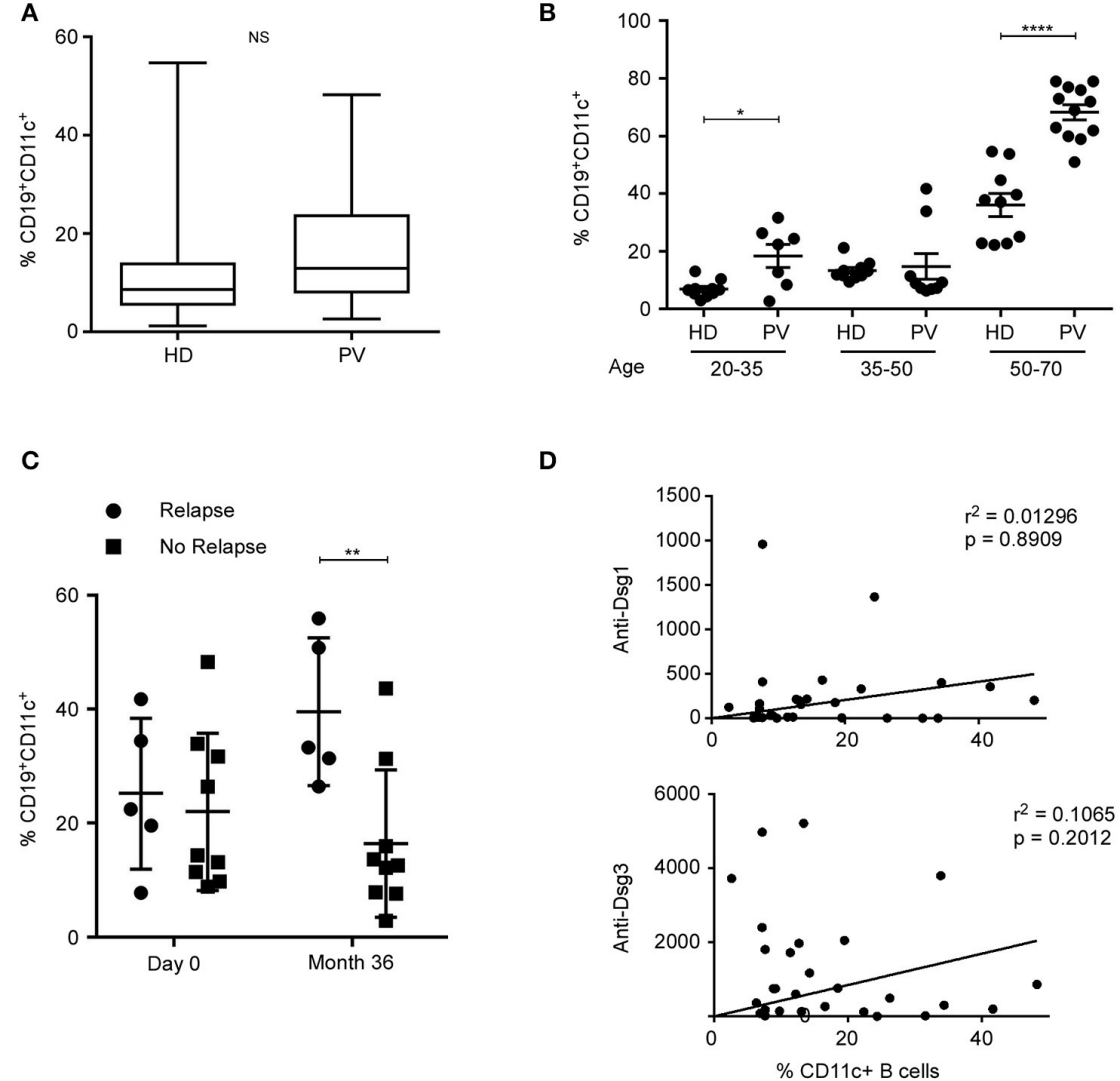
Higher frequency of CD11c^+^ B cells in pemphigus patients with active disease. **(A)** Box plot of CD11c^+^CD19^+^ frequency determined by cytometry in blood of healthy donors (HD) and pemphigus vulgaris (PV) patients. NS, non-significant. CD11c^+^ B cells' frequencies were measured for 28 patients with PV and 52 HD, independently of their age. There was no significant difference between patients with an active autoimmune disease and a large group of HD. **(B)** Percentage of CD11c^+^ B cells for HD and PV donors between age 20 and 35, 35 and 50, and 50 and 70 years old were compared. Significant difference is determined by one-way ANOVA with correction by Bonferroni's multiple comparison test. *p < 0.05, ****p < 0.0001. **(C)** Percentage of CD11c^+^ CD19^+^ B cells was measured by flow cytometry in PV patients at day 0 and 36 months after treatment. Patients were grouped according to whether or not they relapsed after treatment. Means were compared by two-way ANOVA with correction by Sidak's multiple comparison test: **p < 0.01. **(D)** Increase in CD11c B cells does not correlate to anti-DSG titer in PV patient blood (*r*, Pearson correlation coefficient; *n* = 28).

CD11c^+^ B cells have been associated with autoimmune diseases (9–12). Pemphigus is an autoimmune disease affecting skin and mucous membranes, with pathogenic autoantibodies directed against desmogleins 1 and 3 (Dsg1 and Dsg3). We therefore evaluated the frequency of CD11c^+^ B cells in patients with active pemphigus vulgaris (PV) in comparison to HD. Patients with active PV have not a significant higher frequency of CD11c^+^ B cells (*p* > 0.05) than HDs (Figure 8A). However, patients' age ranges from 19 to 79 years old, and Figure 1E shows a significant difference in CD11c^+^ B cells between age groups. We therefore compared CD11c^+^ B cells per age groups and found that patients with active PV have a significant higher frequency of CD11c^+^ B cells than HD in age groups 20–35 and 50–70, but not for the age group 35–50 (Figure 8B). We recently showed that first-line Rituximab treatment allowed achieving an 89% rate of complete remission off therapy at month 24 in pemphigus patients (29). Patients were then evaluated at month 36 when B cells were detectable in blood. PV patients who relapsed have a significant higher frequency of CD11c^+^ B cells than patients in complete remission at month 36 (Figure 8C). Interestingly, the frequency of CD11c^+^ B cells was 1.6 times higher in the relapse groups at month 36 when compare to baseline at day 0 (mean, 25 ± 6% at day 0 vs. 40 ± 6% at month 36, *n* = 5), while in the group of PV patients with no relapse, the frequency of CD11c^+^ B decreased at month 36 when compare to baseline at day 0 (mean, 22 ± 5% at day 0 vs. 16 ± 4% at month 36, *n* = 9). Therefore, CD11c^+^ B-cell frequency increases during active disease in PV patients but decreases after healing. We did not observed a correlation between the percentage of CD11c^+^ B cells and the autoantibodies, anti-Dsg1 and anti-Dsg3 titer (Figure 8D).

## Discussion

An unusual B-cell subset characterized by CD11c expression has been described to be enriched in patients with different autoimmune diseases as RA (9), Sjogren's syndrome (10), multiple sclerosis (11), and SLE (12), as well as during infections or in aged individual (9, 13). However, these B cells can be found in HD as well. This broad range of condition could explain why markers used to characterize this population are diverse. Indeed, a B cell population that expressed Fc receptor-like 4 and CD11c was described in tissue and tonsil of HD, as memory B cells but CD27^−^ (30). Later, Huifang Li described these so called tissue-like-memory B cells in HD blood as FcRL5^+^, CD21^low^, CD27^−^, and CD11c^+^ B cells (15). In our study, we focus our analysis on CD19^+^CD11c^+^ B cells, which include subpopulation expressing FcRL5 or CD21^low^ or CD27^−^ CD11c^+^ B cells, already described. Immunophenotyping, molecular, and functional analyses demonstrated in a consistent manner that CD11c^+^ B cells correspond to a subset of B cells, which mainly but not exclusively belong to memory B cells, and have a strong ability to differentiate into antibody-secreting cells.

We first confirmed that the percentage of CD11c^+^ B cells increased with age in HD (Figure 1E), as reported in mice in which CD11c^+^ B cells accumulate and are known as ABC (9). Despite the fact that CD11c^+^ B cells have been reported to be increased in women with RA, we did not evidence any relationship with gender in HD. No significant differences were observed between age groups for CD11c^hi^ B cells.

Phenotypic analyses showed that the CD11c^+^ B subpopulation was significantly enriched in switched and unswitched memory B cells, while CD11c^−^ B cells were principally naive or DN B cells (Figure 1C), thus confirming Rubtsov's but not Wang's data (9, 12). However, by following Wang's gating strategy on CD19^hi^CD11c^hi^ cells, we observed a significant decrease in switched memory and a significant increase in unswitched memory and naive B cells in comparison with CD11c^+^ B cells. However, the DN B cells were not increased as Wang reported (12). The facts that [1] frequencies of CD11c^hi^ B cells did not change with age while CD11c^+^ B cells increase with age, [2] CD11c^hi^ B cells have a bigger size, and [3] are enriched in the unswitched memory phenotype in comparison with CD11c^+^ B cells strongly suggest that CD11c^hi^ and CD11c^+^ B cells are two distinct populations.

DN cells (CD27^−^IgD^−^) are atypical memory cells, which are reported to be exhausted, anergic, or preplasma cells. The role of DN cells remains unclear, but it has been observed that DN frequency increases during infection (HIV, malaria, Hantavirus), autoimmune disease (SLE, multiple sclerosis), as well as in non-small cell lung cancer (13, 31–35). In addition, DN cells are reported to express CD11c in HIV, malaria, Hantavirus infection, SLE, and multiple sclerosis (11, 36, 37). Based on those observations, one could expect to have a higher frequency of DN cells among CD19^+^CD11c^+/hi^. However, we analyzed HD blood in Figure 1C, and frequency of DN cells was lower in CD19^+^CD11c^+/hi^ than in CD19^+^CD11c^−^ B cells. Our interpretation is that DN CD11c^+^ B cells increase during ongoing immune response, associated with a strong inflammatory response. In addition, SC Jenks shows that DN CD11c^+^ B cells in SLE are poised to generate plasmablasts (33), and J. Fraussen shows that DN cells contain less somatic mutation than switched memory B cells in multiple sclerosis (32). Therefore, we hypothesize that DN CD11c^+^ B cells could be an intermediate step of differentiation toward plasma cells during B-cell activation, while in normal condition, DN CD11c^−^ B cells could be anergic or exhausted B cells.

Despite the preferential expression of CD11c among memory B cells, our phenotypic analyses showed that CD11c was expressed by all B cell subpopulations that we analyzed. To identify a unique surface marker and transcription signature, we performed a microarray analysis on CD11c^+^ and CD11c^−^ B cells purified from five HD.

CD11c^+^ B cells were extremely different from CD11c^−^ B cells with 512 DEG with a fold change above 3. However, none of the cell surface markers identified by microarray was able to strictly discriminate CD11c^+^ from CD11c^−^ B cells by FACS because of their heterogeneous expression (Figures 3C,D). Moreover, CD11c^+^ B cells are often characterized by low or negative expression of CD21 (9, 11). However, gene expression of *CD21* was found downregulated in CD11c^+^ B cells with a fold change <3, and CD21 expression analyzed by FACS revealed that less than 10% of CD19^+^CD11c^+^ were CD21^low/−^ in HD (data not shown). Similarly, IL-21R was reported to be upregulated in SLE patients (12). However, in our study, *IL21R* and *IL21* are not a DEG, and *IL21* was not detected by qPCR, which suggests that these markers are specific of CD11c^+^ B cells in SLE.

Wikipathways and GO analyses identified, in CD11c^+^ B cells, an enrichment of cell activation and cell adhesion molecules, TLR signaling, and inflammatory response. Indeed, upregulation of genes coding for the CD80 and CD86 costimulatory molecules observed in CD11c^+^ B cells was confirmed by FACS for CD86 (Figure 3C). This result is in accordance with studies showing the overexpression of costimulatory molecules in ABCs (9, 11, 12). Interestingly, CD200 was downregulated in CD11c^+^ B cells (Figure 3D), which can inhibit monocyte stimulation through binding to CD200-R (38), while the upregulation of CD58 lead to increased adhesion and activation of T cells through binding to CD2 (39).

TLR7 signaling is crucial for CD11c^+^ B-cell development in mice (40). In addition, TLR9 has been shown to be upregulated in CD11c^+^ B cells from SLE patients (12). Here, we demonstrated that TLR7 or TLR9 stimulation of purified CD11c^−^ B cells was not sufficient to upregulate CD11c expression (Figure 7), whereas BCR stimulation was sufficient. Upregulation of CD11c was reduced as well by almost 2-fold (36.7% vs. 19.6%) by adding CpG on B cells stimulated by anti-IgM + anti-CD40 (12). Therefore, while TLR signaling is an important signaling pathway highlighted by the microarray analysis, TLR signaling does not seem to be crucial for CD11c upregulation in human peripheral B cells.

IRF5 is a transcription factor that can induce *IFN*γ mRNA transcription (41) and regulate IgG class switching in B cells (27, 42). Our qPCR analysis showed an overexpression of *IRF5* by CD11c^+^ B cells (Figure 5D), which is in agreement with a previous report showing that IRF5 participates in the development of ABCs in systemic autoimmunity in mice (43). Activation of TLR7 and TLR9 induces IRF5 translocation in the nucleus, leading to the transcription of IFN and other pro-inflammatory cytokine genes (43). Indeed, we observed a significant increase in *IFN*γ gene expression as well as *IL1*β, *IL7*, and *IL12p40* (Figure 5A). On the other hand, transcriptomic and qPCR analyses showed that the gene expression of EBI3, which is a subunit of the cytokine IL-27, was increased in CD11c^+^ B cells (Figure 5A) (44). Interestingly, IL-27 and IFN-γ can induce B-cell intrinsic T-bet expression, which regulates Ig class switching and is required for the production of long-lived antibody-secreting cells (25, 45).

As previously described in SLE patients, gene expression level of *IL6* was decreased and *IL10* was increased in CD11c^+^ compared with CD11c^−^ B cells (Figure 5) (12). Although IL-6 is a pro-inflammatory cytokine (46, 47) and IL-10 has anti-inflammatory effects, both help in B-cell differentiation and survival as well. Moreover, we previously reported that IL-10 secretion following TLR9 and BCR stimulation was not detected by ELISA in CD11c^+^ B cells from HD (14), suggesting that IL-10 might be completely consumed by B-activated cells differentiating into antibody-secreting cells.

Furthermore, we observed an overexpression of *TBX21* (T-bet) in CD11c^+^ B cells, confirming studies that demonstrated that ABC express high levels of CD11c and transcription factor T-bet (48–52). Although the transcription factor T-bet is not usually expressed by B cells (53, 54), several studies demonstrated its role in Ig class switching (55, 56). Moreover, Rubtsov et al. showed that CD11c^+^ T-bet^+^ B cells were more effective in presenting Ag than other B cell subpopulations (57). Consequently, we observed that, upon stimulation, CD11c^+^ B cells were found prone to differentiate into antibody-secreting cells, especially plasmocytes, with a higher frequency of immunoglobulin class switched than CD11c^−^ B cells subpopulation (Figure 6). However, not all CD11c^+^ B cells are T-bet^+^ in SLE patients or HD, and some T-bet^+^ B cells do not express CD11c (12). Moreover, B-cell-intrinsic T-bet deletion in a murine lupus model exerted no impact of CD11c^+^ B cells generation *in vivo* (16).

Finally, we observed that the frequency of CD11c^+^ B cells was higher in PV patients than HD defined per age groups, as previously reported in RA. Moreover, the frequency of CD11c^+^ B cells increased in a group of PV patients with active disease in comparison with a group in complete remission, 36 months after treatment (Figure 8C), suggesting that CD11c^+^ B-cell frequencies could be an indicator of disease activity. During active disease, CD11c^+^ B cells were enriched in the memory compartment (switched, unswitched, and DN), and we did not observed a preferential expansion of DN cells (data not shown). In addition, we did not observed a correlation between the frequency of CD11c^+^ B cells in the blood and the autoantibodies titer as previously reported in SLE (12), which could be due to the skin localization of B cells in the skin lesions of PV patients (58).

In conclusion, our study has characterized CD11c^+^ B cells in the blood of HD. CD11c is expressed in many B cell subpopulations but is enriched in memory B cells, which have a strong ability to differentiate into plasmocytes and secrete Ig. Accumulation of CD11c^+^ B cells, which naturally occurs with age, could thus promote the emergence of autoimmunity.

## Data Availability Statement

The datasets generated for this study can be found in the National Center for Biotechnology Information's Gene Expression Omnibus (https://www.ncbi.nlm.nih.gov/geo/). Data are accessible using the following accession number: GSE112515.

## Ethics Statement

The studies involving human participants were reviewed and approved by Ethics Committee of the North West in France. The patients/participants provided their written informed consent to participate in this study.

## Author Contributions

M-LG and SC wrote and conceived the manuscript. M-LG, SC, MD, CD, GR, and MM-V generated and analyzed data. OB and PJ read and revised the manuscript.

### Conflict of Interest

The authors declare that the research was conducted in the absence of any commercial or financial relationships that could be construed as a potential conflict of interest.

## Supplementary Material

Supplemental Table 1 List of DEG.

## Abbreviations

ABC: age-associated B cells
DEG: differentially expressed genes
HD: healthy donors
PV: pemphigus vulgaris
RA: rheumatoid arthritis
SLE: systemic lupus erythematosus.
